# The spatial correlation of economic institutional change in China and its impact on economic growth: A social network analysis approach

**DOI:** 10.1371/journal.pone.0297354

**Published:** 2024-10-22

**Authors:** Wenhan Jia, Qianbin Di, Xiaolong Chen

**Affiliations:** 1 School of Geographical Sciences, Liaoning Normal University, Dalian, China; 2 Marine Economies and Sustainable Development Research Center, Liaoning Normal University, Dalian, China; Guangzhou Institute of Geography, Guangdong Academy of Sciences, CHINA

## Abstract

Economic institutional change is a vital driving force behind the rapid rise of China’s economy. However, the incremental approach to economic institutional change has caused unbalanced transformation and economic growth. To this end, we adopted the entropy method to measure the economic institutional change index, and employed social network analysis to reveal its spatial correlation characteristics. We then applied QAP analysis to empirically demonstrate the impact of China’s economic institutional change on regional disparities in economic growth. The findings indicated a gradual increase in the level of economic institutions over time and a spatial gradient between the eastern, central, and western regions. Moreover, the spatial correlation network of China’s economic institutional change is stable and gradually improving. Nevertheless, the role of provinces in the process of economic institutional change varies: the eastern coastal provinces play a dominant role, the central and western provinces benefit to a lesser extent, and some provinces in northeastern China play a “bridging” and “intermediary” role. Regional differences in China’s economic institutional change have widened the regional disparities in China’s economic growth, and the impact of each dimension of economic institutions on regional disparities in economic growth is characterized by phases.

## Introduction

Since the introduction of China’s reform and opening up policy, its economy has leapfrogged into being the world’s second largest. Its GDP per capita rose from 423 yuan in 1979 to 85,698 yuan in 2022, achieving the “Chinese miracle” of economic growth. This rapid economic rise can be attributed to economic institutional change, through which the associated “institutional dividend” brought development opportunities [1, 2]. Throughout the process of economic institutional change over the past 40 years, China has undergone a series of incremental and systematic institutional innovations as well as institutional and structural evolution to liberate and develop its productive forces and successfully transition from a socialist planned economy to a socialist market economy [3].

China’s economic institutional changes are clearly market oriented: the power of control, investment, and distribution of resources and economy gradually transitioned from government to market regulation [4]. A major step in China’s economic transformation is the adjustment of the property rights system, which features the restructuring of state-owned enterprises and encouraging the development of a non-state-owned economy, thereby promoting the diversification of the property rights system and economic growth [5]. The widening and deepening of China’s opening up has broken down barriers to trade liberalization, while foreign trade and investment have provided new opportunities for economic transformation and development [6, 7]. The core of economic institutional change lies in the reform of the distribution system, which includes establishing and improving a distribution system, which sets in place incentive mechanisms, balances or accommodates efficiency and fairness, and forms a new income distribution structure [8]. Specifically, the success of China’s economic institutional change owes much to the incremental change approach, that is, adopting the unbalanced (also known as non-uniform or uneven) strategy to achieve economic institutional change by piloting it in some areas and then promoting it to wider areas [9, 10]. The approach’s spatial and temporal dispersal of the contradictions and conflicts of interest in economic institutional innovation provide a buffer zone for resolving risks in economic institutional innovation [11].

However, the incremental approach to economic institutional change, mostly province and region based, has created a spatial and temporal sequence in the institutional and policy environment for unbalanced transformation in China, which has also indirectly affected its spatial pattern of economic development [12]. Furthermore, the long-term neglect of multiple spatial effects in the process of economic institutional change has affected China’s balanced economic growth and regional economic development [13]. Against this backdrop, this study innovatively adopted the spatial correlation perspective to integrate the economic institutional changes in provinces and municipalities across China into a unified overall framework to examine the spatial and temporal characteristics of their economic institutional changes and constitutive networks. This assisted to clarify the status and roles of provinces and municipalities in the network of economic institutional changes in China, which is particularly important for the spatial balance of China’s economic institutions and the construction of a higher level of socialist economic institutions. Moreover, this study extensively investigated the impact of unbalanced economic institutional change on regional disparities in economic growth, which assisted in providing fresh ideas for reducing regional economic disparities and achieving balanced regional economic development.

**Fig 1:**
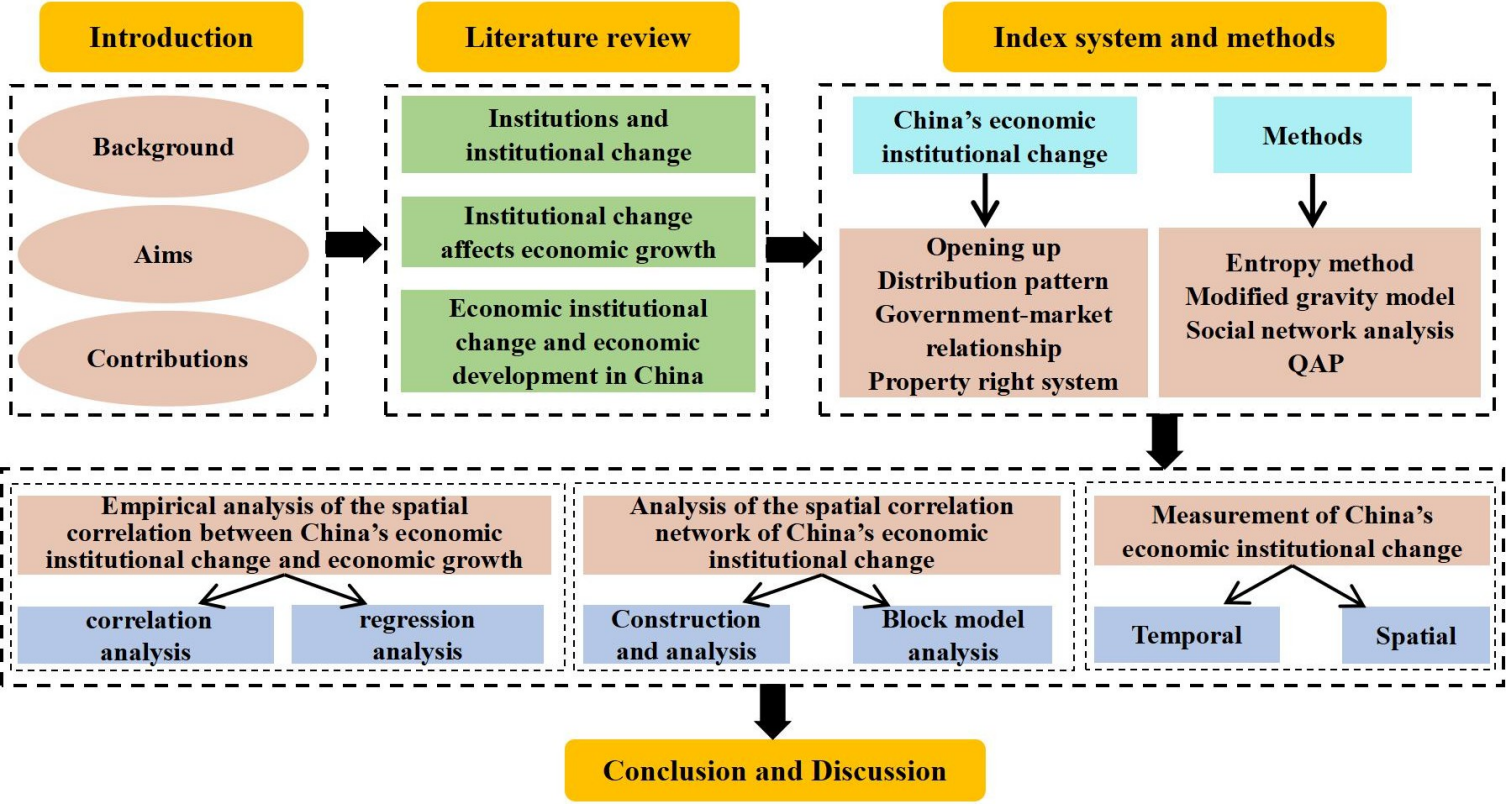
The framework of this article.

The framework of this paper proceeds as follows (Fig 1). The Literature Review section summarizes and reviews the existing literature. The Materials and Methods section introduces the data sources and research methods. The Results section presents our results of this study. The Discussion section is the discussion. The Conclusions section draws the conclusion.

## Literature review

### Institutions and institutional change

The Western institutional school began to study the role of institutional factors in social and economic development early. The old institutional school, represented by Veblen [14], Commons [15] and others, defined the concept of institution broadly in terms of ownership, distributive relations, and legal institutions, among others. The 1950s saw the rise of a new school of institutional economics, represented by Galbraith [16], Coase [17] and others, which redefined institutions as norms of behavior and interactions between people or organizations.

North’s theory of institutional change is an important branch of the new institutional economics stream. North [18] views institutions as a set of norms and social structures that regulate and constrain the behavior of individuals. In his 2001 book, Institutions, institutional change and economic performance, he explained institutional change as the process by which individuals with the ability to negotiate over rules adjust to a comprehensive or integrated institutional framework constructed by formal and informal rules, which impacts economic performance through transaction costs. In addition, the theory of evolutionary institutional change [19] and the theory of institutional change from the evolutionary game view [20] are the main analytical frameworks for institutional change in the West. The driving force of institutional change, there are two explanations of endogenous change and exogenous change, exogenous change refers to new knowledge, the emergence of new technologies will change the external environment, resulting in institutional imbalances to generate new institutional needs, when the expected benefits of the new institutional arrangements are higher than transaction costs of institutional change when institutional change has occurred [21]. Endogenous change that the intrinsic characteristics of the institutions induces its own change, the institutions of self-reinforcement, self-weakening corresponds to the system of institutional stabilization, system of institutional change in two phases, respectively [22, 23].

### Institutional change affects economic growth

Regarding the relationship between institutional change and economic growth, North [24] established a theoretical framework to investigate economic growth. In The rise of the Western world, North and Thomas [25] pointed out that institutional change determines economic growth by influencing the motivation of economic entities and the efficiency of economic organizations. Transactions, as the smallest structural unit of an economic institutions, are the basis for comparative analysis of institutions, and institutional change acts on economic growth through transaction costs [26]. At the same time, there are differences in the impact of different dimensions of institutions on economic growth, with institutional flexibility contributing more significantly to economic growth [27]. Easterly and Levine [28] as well as Rodrik et al. [29] and Acemoglu et al. [30] employed regression analysis to demonstrate, empirically, the impact of institutional factors on the efficiency of economic growth based on cross-country data.

In addition to the micro-paths of institutional change and economic growth, researchers also focus on their spatial effects. Institutions exhibit a spatial spillover effect in development, that is, the level of institutions between countries and regions displays a mutual influence [31]. Additionally, institutions not only influence local economic growth, but also affect the economic growth of surrounding regions through spillover effects [32, 33]. Moreover, some scholars have associated institutional change with “space of flows,” a factor that affects economic growth. Lothian [34] and Alfaro et al. [35] associated institutions with capital flows to investigate the impact of institutions on cross-national capital flows. Krammer [36] and Vargo et al. [37] analyzed the relationship between institutions and the spatial spillover effects of technological innovation, confirming the impact of institutions on technological innovation flows. Sun et al. [38] empirically demonstrated the role of institutional soft environment on regional talent attraction, taking the impact of institutional distance on talent flow patterns and cross-regional mobility in China as an example.

### Economic institutional change and economic development in China

Since the 1970s, China has carried out economic reforms, opened up to the outside world, encouraged the development of the non-state economy, and gradually shifted from a planned economic system to a market economic system, which is in essence a process of economic institutional change, and has greatly contributed to China’s economic growth [39, 40]. At the same time, China’s economic institutional change process is also characterized by gradualism, lagging and path dependence [41, 42].

In terms of economic institutions measurement, the entropy method and principal component analysis are often adopted to obtain institution proxy variables to characterize China’s economic institutional change [43, 44]. In the study of the relationship between economic institutional change and economic growth in China, scholars at home and abroad have also achieved a lot of results. Two schools of thought—the “experimental school” and the “convergence school”—are popular among foreign scholars who explain China’s economic growth from the perspective of economic institutional change. Nevertheless, both focus on the role and impact of different perspectives of economic institutional change on economic growth [45]. Liu [46] and Qiang and Jian [47] quantified institutional factors and empirically analyzed the impact of China’s institutional change on economic growth from the degrees of market resource allocation, market openness, and diversification of property rights. Zhang and Wang [48] analyzed the impact of economic institutional change on China’s economic growth in the context of the country’s high-quality economic development goal at the current stage. Moreover, the issue of uneven economic institutional change and economic development in China has received extensive attention. Young [49] analyzed the reform process led to the fragmentation of the domestic market and the distortion of regional production away from patterns of comparative advantage, while Huang [50] proved that economic institutions are a key influencing factor of regional economic disparities in China.

## Materials and methods

### Data sources

Considering the consistency of statistical data and caliber, as well as the impact of COVID-19 on statistical data in 2019, the study time interval from 1997 to 2018 was selected. And 31 provinces and municipalities in China were selected as the study area units. The data in this paper are from China Statistical Yearbook and statistical year-book of China’s province and municipalities from 1996 to 2019.

### Index system construction

Drawing on the research outcomes of previous studies and considering the connotation and trend characteristics of China’s economic institutional change, we constructed a system of indices for evaluating China’s economic institutional change from the opening up, distribution pattern, government-market relationship, and property rights system perspectives [51], as shown in Table 1.

**Table 1:** System of indices for evaluating China’s economic institutional change.

First-level	Second-level	Third-level	Attribute	Weight
Opening up	Openness to foreign trade	Value of international trade/GDP (%)	+	0.112
	Openness to foreign investment	Foreign funded/Investment in fixed assets (%)	+	0.107
Distribution pattern	Distribution	Disposable income of urban households/GDP (%)	+	0.115
	Redistribution	General public budget revenue/GDP (%)	+	0.098
	Equity	Disposable income of urban households/Disposable income of rural households (%)	-	0.095
Government-market relationship	Marketization	Marketization index^a^	+	0.095
	Government intervention	General public expenditure/GDP (%)	-	0.094
		Tax revenue/GDP (%)	-	0.095
Property right system	Denationalization	Output value of Non-state-owned industrial /Gross industrial output value (%)	+	0.094
		Employed person of non-state-owned units/Employed person in urban areas (%)	+	0.096

^a^Marketization index is derived from《NERI INDEX》compiled by Fan and Wang, and some missing data are derived from the annual growth rate.

### Methods

**Fig 2:**
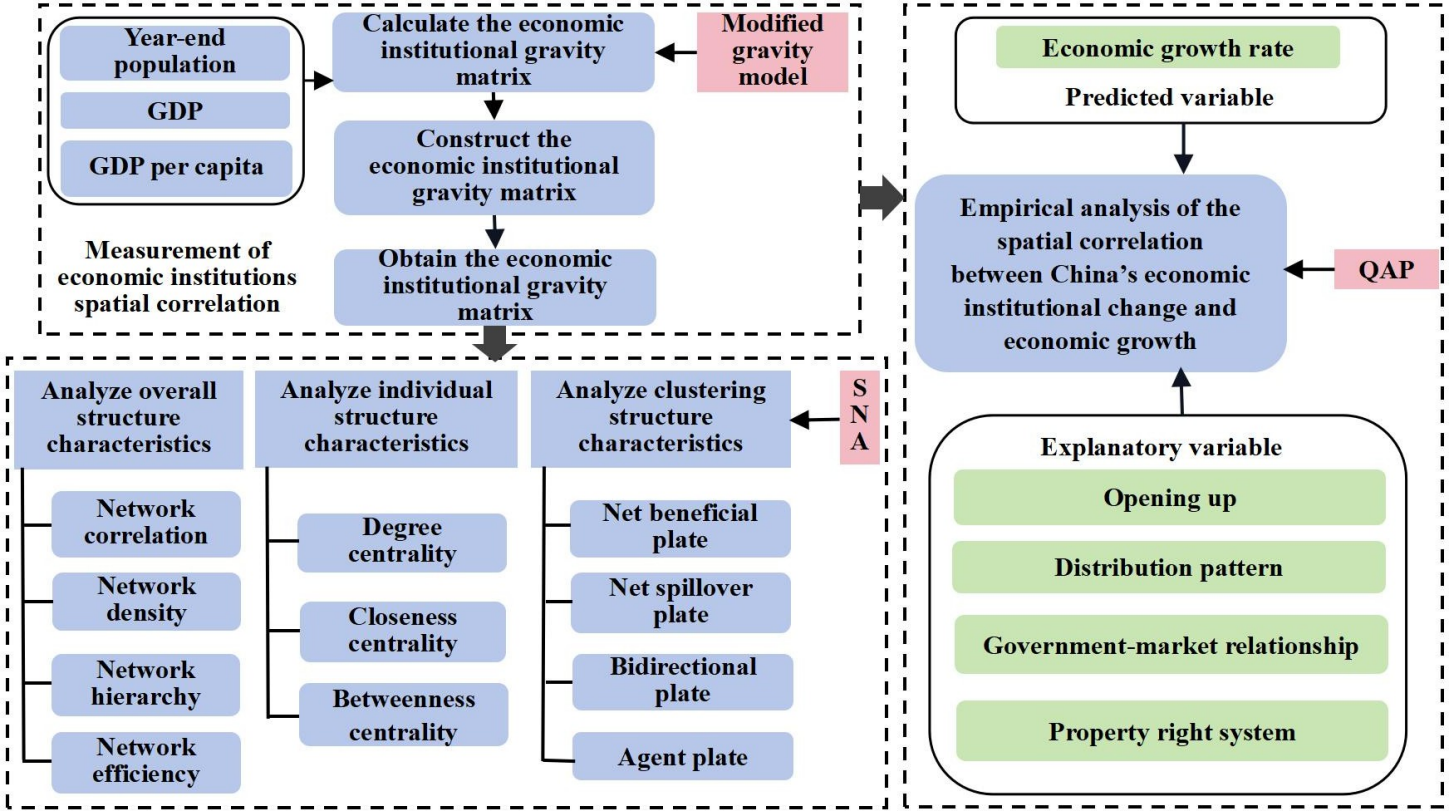
Research logic diagram.

The logic of this research is shown in Fig 2. The specifics of the methods for each step are listed below.

#### Entropy method

Entropy method is an objective weighting method commonly used in the comprehensive evaluation of index system, and the weight of each index is determined by objective difference degree. This method can more objectively and effectively measure the economic institutional changes of provinces and municipalities in China. Since entropy method is a common objective weighting method, it will not be introduced here.

#### Modified gravity model

The modified gravity model is based on the law of universal gravity and gravity model, and proposed by Tinbergen and Pobyhobnen after considering the developmental characteristics, laws and influencing factors in the field of economic research [52, 53]. It is widely used in the calculation of interaction between cities and regions and the determination of spatial correlation.


$$R_{ij}=L_{ij}\frac{\sqrt[{3}]{G_{i}P_{i}I_{i}}\sqrt[{3}]{G_{j}P_{j}I_{j}}}{{\left(\frac{D_{ij}}{g_{i}-g_{j}}\right)}^{2}},L_{ij}=\frac{I_{i}}{I_{i}+I_{j}}$$ (1)

Where *R*_*ij*_ represents the gravitational strength between province *i* and *j*, *L*_*ij*_ represents the gravitational coefficient between province *i* and *j*. *G*_*i*_ and *G*_*j*_ are the GDP of provinces *i* and *j* respectively, *P*_*i*_ and *P*_*j*_ are the permanent population of province *i* and *j* respectively, *I*_*i*_ and *I*_*j*_ are the economic institutional change indexes of provinces *i* and *j* respectively. *D*_*ij*_ indicates the distance between provincial capitals, *g*_*i*_ and *g*_*j*_ are the per capita GDP of province *i* and *j* respectively.

#### Social network analysis

Social network analysis is an analytical method used to describe and analyze the relationship characteristics and types of social things and their relationship networks through relational data, including overall network analysis and individual network analysis [54, 55]. Its various index, formulas and meanings are shown in Table 2.

**Table 2:** Description of indicators of social network analysis.

Index	Formulas	Meanings
Network density	D=L/[N×(N−1)] *D* is the network density, *L* is the number of existing linkages, *N* is the total number of linkages.	The network density reflects the degree of network correlation.
Network connectedness	C=1−V/[N×(N−1)/2] *C* is the network connectedness, *V* is the number of unreachable pairs of points in the network.	The network connectedness represents the robustness of network structure.
Network hierarchy	H=1−K/max(k) *H* is the network hierarchy, *k* is the number of symmetrically reachable pairs of points in the network.	The network hierarchy reflects the dominant position of the network.
Network efficiency	E=1−M/max(M) *E* is the network efficiency, *M* is the number of redundant lines in the network.	The network efficiency represents the overflow paths in the network.
Degree centrality	DC=n/(N−1) *DC* is the degree centrality, *n* is the direct correlation number.	The degree centrality reflects the control ability of individual in the network.
Closeness centrality	$cc=\sum_{j=1}^{N}d_{ij}$ *CC* is the closeness centrality, *d*_*ij*_ is the shortest distance from province *i* to province *j*.	The closeness centrality presents the association and cooperation in the network.
Betweenness centrality	$BC=\frac{2\sum_{j}^{N}\sum_{k}^{N}b_{jk(i)}}{N^{2}-3N+2}{,b}_{jk}(i)=\frac{g_{jk(i)}}{g_{jk}}$ *BC* is the betweenness centrality, *g*_*jk(i)*_ is the number of connections passing through region *i* out of all connections from province *j* to province *k* in the interior.	The betweenness centrality reflects the intermediary status of individual in the network.

#### QAP

Based on the replacement of matrix data, quadratic assignment procedure explores the relationship between corresponding elements in two or more matrices, and obtains the test method of matrix correlation and regression [56, 57]. This method can effectively solve the problems of multicollinearity and false correlation.


$$\Omega =f(X_{1},X_{2},\cdots X_{n})$$ (2)

Where *Ω* represents the spatial network relation matrix of the research object, *X*_*m*_ (*m* = 1,2,⋯,*n*) represents the influencing factor matrix.

## Results

### Measurement of economic institutional change in provinces and municipalities across China

Based on the index system constructed above and the index weights derived from the entropy method, we measured the index of economic institutional change in provinces and municipalities across China.

**Fig 3:**
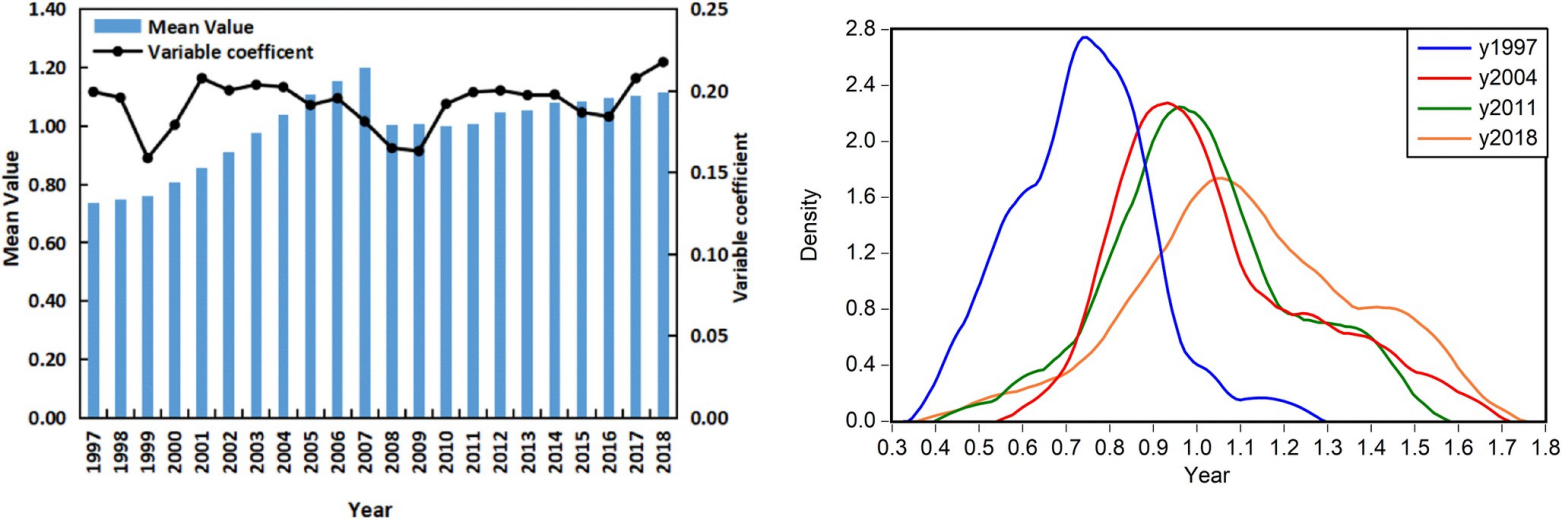
Temporal distribution of China’s economic institutional change (a)Descriptive statistics (b)Nuclear density.

**Fig 4:**
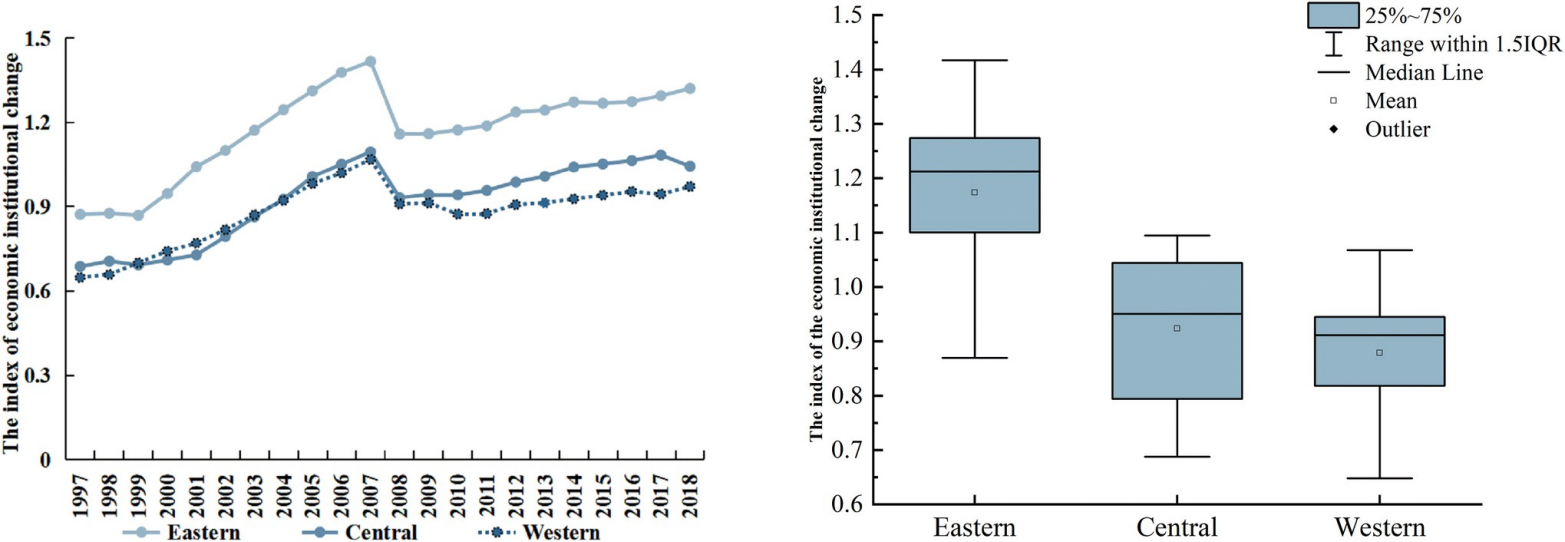
Analysis of regional differences in China’s economic institutional change.

As can be observed from Figs 3 and 4, the temporal and spatial distribution of economic institutional change in China is uneven and exhibits significant differences. From a temporal perspective, the mean value increases from 0.738 to 1.114, the index of China’s economic institutional change generally exhibits an increasing trend: economic institutional change was slow before 2000, rapid from 2000 to 2008, and slowly increasing after 2008 owing to the economic crisis. In nuclear density analysis, the main peak of nuclear density curve shifted to the right obviously, the height of the crest decreases and the width of the crest widens from narrow, indicating that the level of China’s economic institutions is increasing, and the regional gap in the level of economic institutions is expanding continuously. Spatially, the economic institutional change of these regions is uneven and exhibits significant regional differences: the economic institutional change in the eastern region is faster than in the central and western regions. Guangdong, Shanghai, Zhejiang, Beijing, and Jiangsu rank among the top provinces and municipalities, while the economic institutional change in the western provinces and municipalities such as Tibet, Qinghai, and Xinjiang rank lower than others (S1 Fig and S1 Table).

### Analysis of the spatial correlation network of China’s economic institutional change

#### Construction and analysis of the spatial correlation network

**Fig 5:**
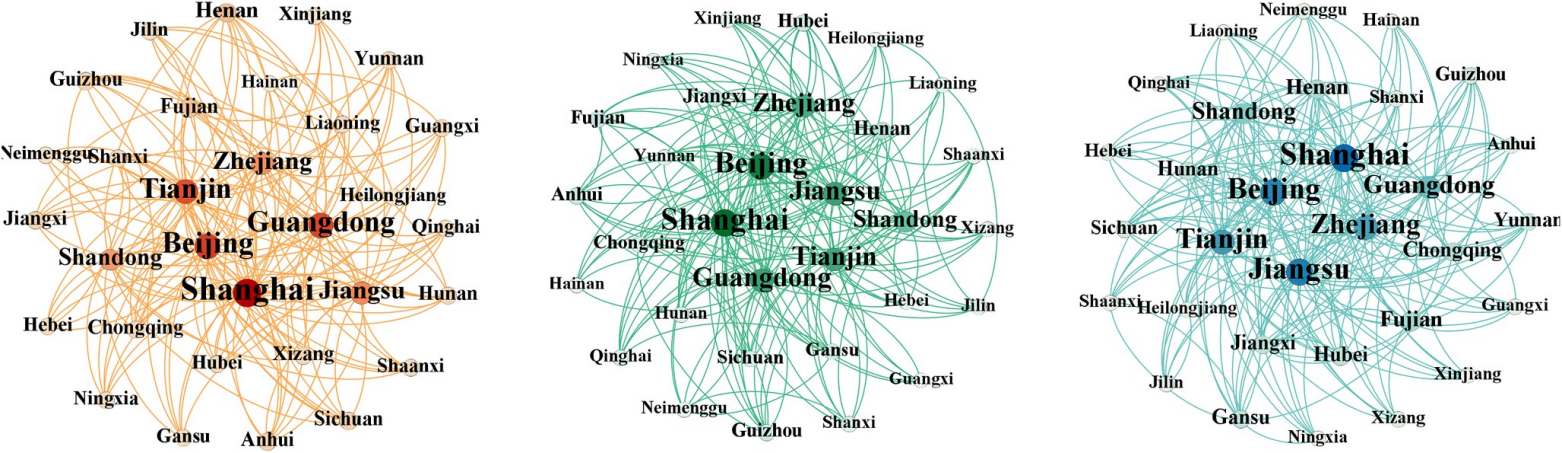
The spatial correlation network of China’s economic institutional change (a)1997 (b)2007 (c)2018.

We employed the modified gravity model to construct a spatial spillover relationship matrix for China’s economic institutional change and used Gephi to select part of the cross-sectional data in order to construct a spatial correlation network (S2 Fig). As shown in Fig 5 and Table 3, the spatial correlation network of China’s economic institutional change is relatively stable, and its network density is increasing but remains relatively low. The network relevance is constantly at 1, implying that no provinces and municipalities are in isolated development. The network rank is in a fluctuating state, and the recent upper limit (with the exception of 1999) is also stable at around 0.95. The network efficiency value and network spillover pathway are increasing and the network stability is gradually improving. Beijing, Shanghai, Jiangsu, Guangdong, Zhejiang, and Tianjin are strongly correlated with other provinces and municipalities and play a strong dominant and controlling role in the network. In contrast, the western and central provinces and municipalities are less correlated and are in a weaker position in the network owing to their level of economic development and geographical location. However, the correlation between them gets stronger over time, and their position and role in the overall network gradually improve.

**Table 3:** Network density and spatial correlation analysis.

Year	Network density	Correlation analysis			
		Network connectedness	Network hierarchy	Network efficiency	Nearest upper limit
1997	0.191	1	0.458	0.738	0.924
1999	0.186	1	0.533	0.747	0.881
2001	0.180	1	0.463	0.761	0.924
2003	0.181	1	0.378	0.759	0.952
2005	0.199	1	0.378	0.729	0.952
2007	0.211	1	0.380	0.708	0.954
2009	0.229	1	0.335	0.683	0.968
2011	0.239	1	0.341	0.669	0.977
2013	0.246	1	0.466	0.653	0.952
2015	0.241	1	0.459	0.664	0.938
2017	0.237	1	0.285	0.676	0.979

**Fig 6:**
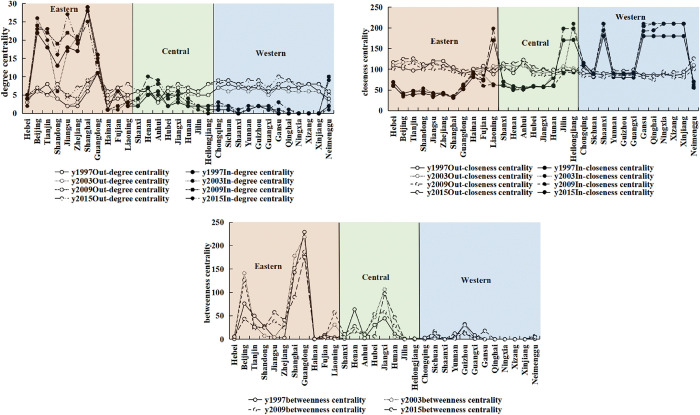
The analysis of centrality of provinces and municipalities across China (a)degree centrality (b)closeness centrality (c)betweenness centrality.

Centrality is a quantification of the position of China’s provinces and municipalities in the spatial correlation network of economic institutional change. Fig 6 indicates the degree, closeness, and betweenness centrality of China’s provinces and municipalities. According to the figure, the spatial correlation networks of China’s economic institutional change generally exhibit more receiving relationships than spillover relationships, and the spillover effect is weak. The node connection pathway is relatively unitary, with strong reliance on intermediate cities. With a relatively high in-degree centrality and a strong factor adsorption capacity, the eastern region is in a dominant and leading position in these networks. With a high closeness centrality and a short distance from other provinces and municipalities in the network, Jilin, Heilongjiang, Shaanxi, Gansu, Qinghai, Ningxia, Tibet, and Xinjiang are “central actors” in the spatial correlation network. Beijing, Shanghai, and Guangdong, given their high betweenness centrality and more receiving and spillover relationships, play a strong controlling role in the spatial correlation of other provinces and municipalities, and are key nodes in the network (S2 Table).

#### Block model analysis

**Fig 7:**
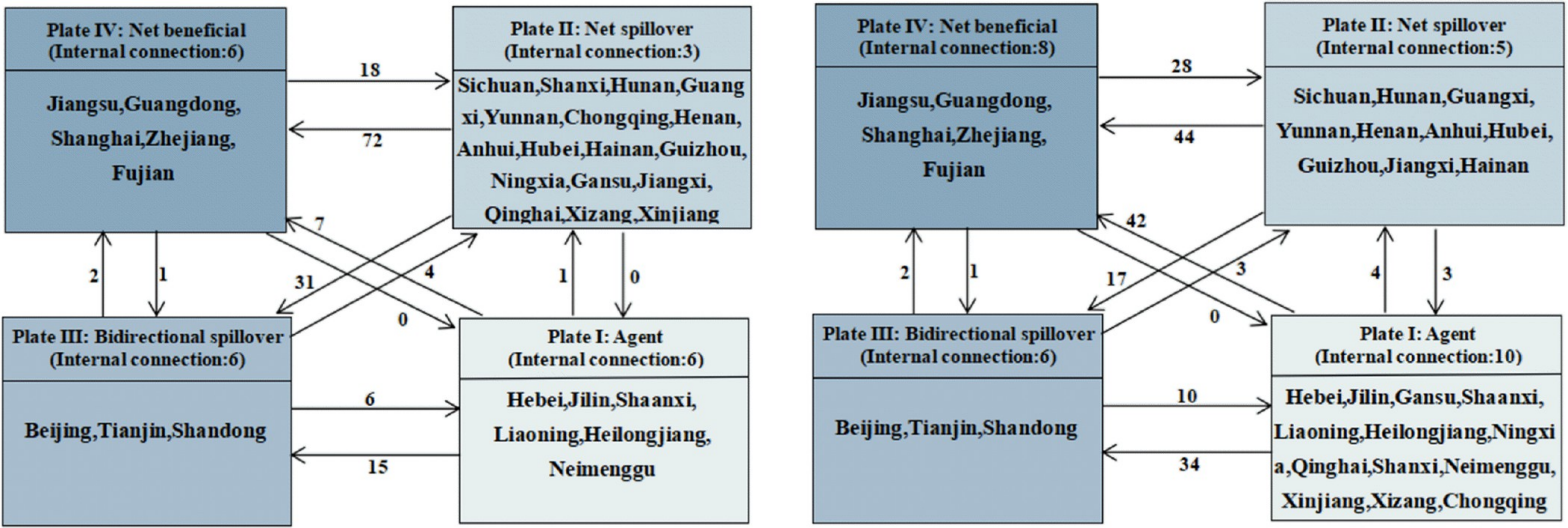
Spillover effect of spatially related plates of China’s economic institutional changes (a)1997 (b)2018.

To reveal the spatial clustering characteristics of the spatial correlation network of China’s economic institutional change further, we utilized the block model to divide the status of China’s 31 provinces and municipalities in the network into four major segments (S3 Table), as shown in Fig 7. According to the block model analysis, the status and role of Chinese provinces and municipalities in the spatial correlation networks differ significantly. The “agent” segment mainly covers the northeastern and central provinces, whose geographical location and development level enable them to play a bridging and linking role in the network. The “net spillover” segment mainly covers some provinces and municipalities in the central and western regions, which exhibit more spillover relationships than receiving relationships, and whose external economic dependence is high. The “bidirectional spillover” segment is mainly composed of Beijing, Tianjin, and Shandong. The “net beneficial” segment is mainly composed of Jiangsu, Shanghai, Guangdong, Zhejiang, and Fujian, mainly involving the eastern coastal provinces and municipalities, with high levels of economic development and strong attractiveness to factors from other provinces and municipalities.

In sum, the provinces and municipalities on the eastern coast of China, which have faster economic institutional change, are still in a stage where the polarization effect is greater than the trickle-down effect. They have a stronger attraction to factors from other regions, exhibiting evident polarization. That is, they are in a dominant position in the spatial correlation network of economic institutional change. Central and western provinces and municipalities, where economic institutional change is slower, exhibit more spillover relationships than receiving relationships, and exhibit a productive factor spillover effect. They are at a disadvantage in the spatial correlation network.

### Empirical analysis of the spatial correlation between China’s economic institutional change and economic growth

#### Model setting and explanation of variables

Starting with the four dimensions of economic institutional change, we employed QAP analysis in UCINET to probe the impact of the spatial imbalance of economic institutional change on regional disparities in China’s economic growth. We set up the following models to probe the impact of economic institutional changes on regional disparities in economic growth:

EGR=f(OU,DP,GMR,PRS) (3)

Based on the measurement of the index of economic institutional change, the difference matrix of opening up (*OU*), distribution pattern (*DP*), government-market relationship (*GMR*) and property rights system (*PRS*) was used as the explanatory variable, and the difference in economic growth rate (*EGR*) of each province and municipalities was used as the predicted variable. The difference matrix of the explanatory variables and the predicted variables are 31×31, taking the *EGR* as an example, *EGR*_*ij*_ represents the difference in the economic growth rate between province *i* and *j*. In order to avoid the impact of dimensional inconsistency on the research results, the average value of the difference matrix is used to standardize the variables.

#### QAP correlation analysis

We employed UCINET to perform QAP correlation analysis on the matrix of differences between the economic institutions’ dimensions and the matrix of differences between the provinces and municipalities’ economic growth rates. The results in Table 4 indicate that regional differences in opening up and distribution patterns are positively correlated with regional disparities in economic growth for all periods, and this correlation is significant at the 1% level. The correlation coefficients are 0.191 and 0.383, respectively. Despite this significant correlation, there is an issue of co-linearity between the explanatory variables, and therefore QAP regression analysis is required.

**Table 4:** The results of QAP correlation analysis.

	Economic growth rate	Opening up	Distribution pattern	Government-market relationship	Property right system
Economic growth rate	1.000***	0.191***	0.383***	-0.060	0.061
Opening up	0.191***	1.000***	-0.193***	0.626***	0.358***
Distribution pattern	0.383***	-0.192***	1.000***	-0.531***	-0.402***
Government-market relationship	-0.060	0.626***	-0.531***	1.000***	0.769***
Property right system	0.061	0.358***	-0.402***	0.769***	1.000***

Notes: * p < 0.1, ** p < 0.05, *** p < 0.01.

#### QAP regression analysis

Based on the correlation analysis above, we employed QAP regression analysis to explore the influential relationships between the variables. From Table 5, it can be observed that regional disparities in terms of opening up, distribution pattern, government-market relations, and property rights system are the primary institutional factors affecting regional disparities in economic growth across provinces and municipalities in China during the 1997–2018 period, with standard regression coefficients of 0.353, 0.417, -0.337 and 0.362 respectively. In terms of opening up, distribution pattern, government-market relations, and property rights system, the regional disparities during this period widened the regional disparities in economic growth, while the widening of regional disparities in government-market relations helped to bridge the regional disparities in economic growth. This is because narrowing the regional disparities in economic growth required the government to adapt to local conditions and implement macro-regulation, thereby making the two exhibit a negative influential relationship.

**Table 5:** The results of QAP regression analysis in full period.

Explanatory variable	Un-Stdized	Stdized Coef	P-value	As Large	As Small
Opening up	0.134	0.353	0.002	0.002	0.999
Distribution pattern	0.132	0.417	0.001	0.001	1.000
Government-market relationship	-0.036	-0.337	0.025	0.975	0.025
Property right system	0.241	0.362	0.001	0.001	1.000

Owing to the long time-interval of the full-period regressions and the crude results of the analysis, we conducted year-by-year regression analysis. As shown in Table 6, the impact of regional disparities in various dimensions of economic institutions on regional disparities in economic growth is complicated and characterized by phases. From 1997 to 2003, regional disparities in China’s economic growth were mainly caused by regional disparities in opening up and the property rights system. Since this was a period of in-depth and comprehensive reform and opening up developments, when denationalization was in full implementation, opening up and the property rights system in this period had a strong pulling effect on economic growth, causing it to have a significant impact on regional economic growth disparities. In the period 2004–2008, the regional disparity in the property rights system was the main institutional factor affecting regional economic growth disparity. Moreover, during this period, special attention was paid to state-owned enterprise reformations and the establishment of a modern property rights system, and since the denationalization of the property rights system and the development of a multi-ownership economy are long-term processes, their impact on regional economic growth disparities is also continuous. In the 2009–2017 period, regional disparities in distribution patterns and property rights systems were the main institutional reasons for the formation of regional disparities in economic growth, and apart from the impact of property rights systems, regional disparities in distribution patterns had a significant impact on regional disparities in economic growth.

**Table 6:** The results of QAP regression analysis year by year.

Year	Opening up	Distribution pattern	Government-market relationship	Property right system
1997	0.030	0.170**	-0.580***	0.647***
1998	0.360***	0.331***	-0.341***	0.139
1999	0.292***	0.169***	-0.675***	0.450***
2000	0.489***	-0.314***	-0.681***	0.535***
2001	1.035***	-0.020	-0.774***	0.077
2002	0.347***	0.078	-0.490***	0.484***
2003	0.335***	-0.284***	-0.715***	0.630***
2004	-0.158*	0.000	-0.199	0.292**
2005	-0.195*	-0.274***	-0.129	0.307**
2006	-0.068	0.259***	0.348**	-0.106
2007	-0.099	0.021	-0.336*	0.476***
2008	0.001	0.030	-0.186	0.001
2009	-0.353***	0.145***	0.192*	0.494***
2010	-0.557***	-0.149**	0.194*	-0.222**
2011	-0.776***	0.073*	0.242**	-0.215***
2012	-0.216**	0.372***	-0.062	-0.021
2013	0.272***	0.449***	-0.246**	0.178**
2014	0.216**	0.435***	-0.136	0.485***
2015	0.289***	0.552***	-0.200*	0.837***
2016	0.064	0.450***	0.066	0.593***
2017	0.090	0.356***	-0.146	0.327***
2018	-0.030	0.529***	0.067	0.095***

Notes

* p < 0.1

** p < 0.05

*** p < 0.01.

In sum, regional disparities in China’s economic institutional changes have widened regional differences in its economic growth. From the economic institutions perspective, to promote balanced and coordinated regional economic development, we need to attach special attention to current regional disparities in property rights systems and distribution patterns, and reduce regional economic growth disparities by promoting a spatially balanced development of property rights systems and distribution patterns.

## Discussion

This article focuses on the spatial-temporal evolutionary characteristics of China’s economic institutional change and its impact on economic growth. Indeed, China’s economic institutional level are constantly improving since China’s economic reform, but the incremental approach to economic institutional change has caused a gradient difference where the economic institutional change in the eastern region is better than in the central and western regions [12]. Therefore, when implementing economic policies in the later stages, greater attention should be given to the status quo of economic institutional development in the central and western regions, as this could help provide better conditions for economic development in the central and western regions.

In addition, institutions exhibit a spatial spillover effect in development, that is, the level of institutions between countries and regions displays a mutual influence [31–33]. We should attach greater importance to the establishment of a spatial correlation network for national-level economic institutional changes while paying attention to local economic institutional changes. Some questions merit further thoughts on, for example, how to give full play to the spillover and pulling effects of the eastern region in the spatial correlation network of economic institutional changes, and how to improve the disadvantaged position of the western region in the spatial correlation network [58]. To solve the uneven regional development and coordination between regional economic development issues, by considering the relationship between regional economic institutions disparities and regional economic growth disparities as the entry point, we should steer towards balanced regional economic development by introducing appropriate changes to the economic institutions [59].

There are still some shortcomings in the article. The article constructed the evaluation index system of the level of China’s economic institutions, but the subsequent selection of indicators can continue to be enriched and perfected. The research scale unit is relatively large in terms of the provincial area, and the subsequent research can be deepened to the level of prefectural cities and counties. The investigation of the spatial correlation between economic institutional change and economic growth needs to be in-depth.

## Conclusions

In addition to measuring the index of economic institutional change, this study applied modified gravity model and social network analysis to examine the characteristics its spatial correlation network. QAP analysis was then performed to probe the impact of spatial imbalance in China’s economic institutional change on regional economic growth disparities.

The findings of this study indicate that (1) There exists spatial and temporal variation in the index of economic institutional change in China. In general, there is a gradual increase from the temporal perspective, and spatially, there exists a gradient difference where the economic institutional change in the eastern region is better than in the central and western regions. (2) The structure of the spatial correlation network of China’s economic institutional change is relatively stable and constantly improving. The “net beneficial” and “bidirectional spillover” segments, primarily in the eastern coastal provinces, play a dominant role in the network, while the “net spillover” segments, mainly in the western and central provinces, benefit to a lesser extent. Further, the “agent” segments, mainly in the northeastern provinces, play a “bridge” and an “intermediary” role. (3) Regional differences in terms of China’s economic institutions changes have affected regional disparities in China’s economic growth. Specifically, first, regional disparity in a property rights system is a long-term and stable driving force for regional economic growth disparity. Second, regional disparity in opening up contributed significantly to regional economic growth disparity in the earlier periods covered by the study. Lastly, regional disparity in distribution patterns is a key influencing factor of regional economic growth disparity in the current stage.

## Supporting information

S1 Fig Spatial distribution of China’s economic institutional change (a)1997 (b)2004 (c)2011 (d)2018.
(TIF)

S2 Fig Spatial linkage strength of economic institutional change in China (a)1997 (b)2018.
(JPG)

S1 Table The economic institutional change of provinces and cities in China. (DOCX)

S2 Table The analysis of centrality of provinces and municipalities across China. (DOCX)

S3 Table Block model analysis. (DOCX)

S1 Data (ZIP)
