# Supplementary figures and images for: The spatial correlation of economic institutional change in China and its impact on economic growth: A social network analysis approach
Source: PLoS One. 2024 Oct 22;19(10):e0297354. doi: 10.1371/journal.pone.0297354 (PMC11495625; doi:10.1371/journal.pone.0297354)

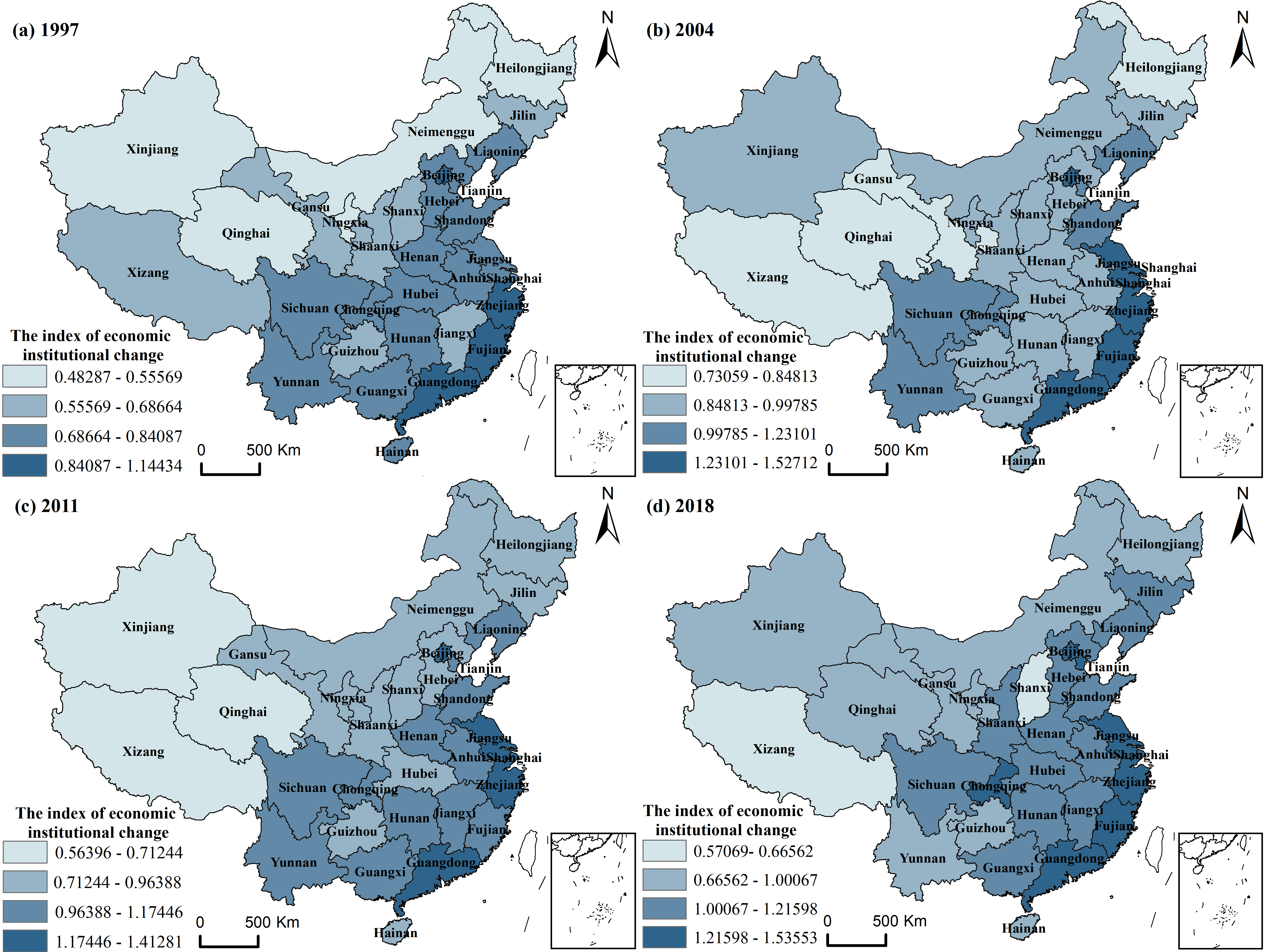

Supplement: S1 Fig — Spatial distribution of China’s economic institutional change (a)1997 (b)2004 (c)2011 (d)2018. (TIF) [file pone.0297354.s001.tif]

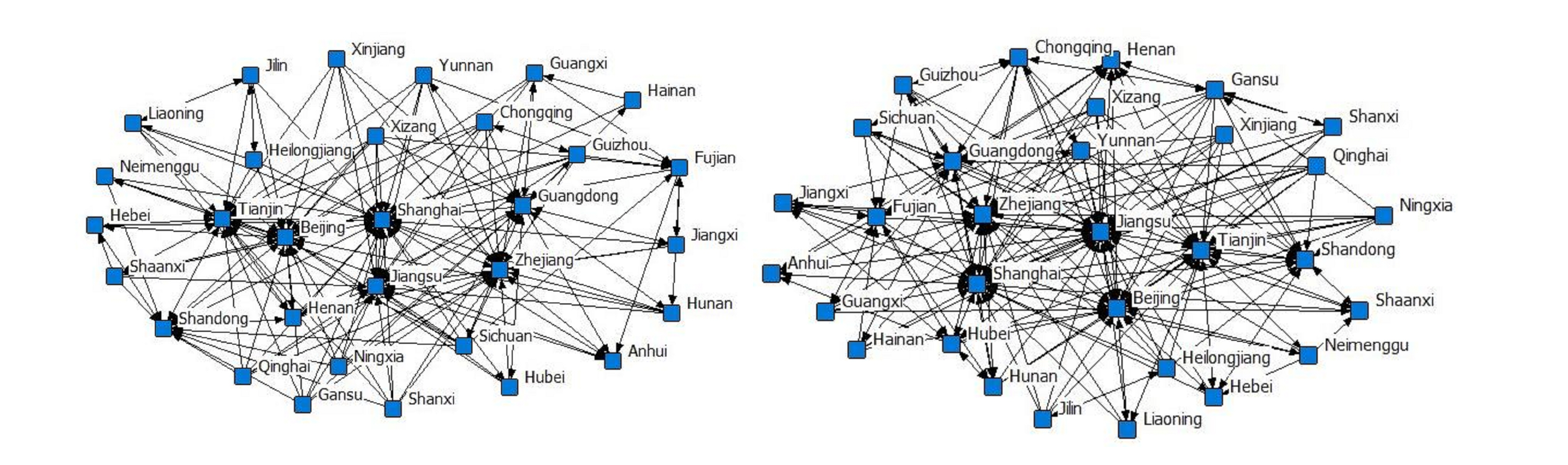

Supplement: S2 Fig — Spatial linkage strength of economic institutional change in China (a)1997 (b)2018. (JPG) [file pone.0297354.s002.jpg]
