# Supplementary material for: The spatial correlation of economic institutional change in China and its impact on economic growth: A social network analysis approach
Source: PLoS One. 2024 Oct 22;19(10):e0297354. doi: 10.1371/journal.pone.0297354 (PMC11495625; doi:10.1371/journal.pone.0297354)
Supplement: S1 Table — (DOCX) [file pone.0297354.s003.docx]

| **Provinces** | **1997** | **2000** | **2003** | **2006** | **2009** | **2012** | **2015** | **2018** |
| --- | --- | --- | --- | --- | --- | --- | --- | --- |
| Hebei | 0.784 | 0.783 | 0.895 | 1.059 | 0.973 | 0.929 | 1.013 | 1.047 |
| Beijing | 0.951 | 0.954 | 1.223 | 1.513 | 1.224 | 1.373 | 1.369 | 1.447 |
| Tianjin | 0.820 | 0.960 | 1.133 | 1.365 | 1.093 | 1.291 | 1.311 | 1.430 |
| Shandong | 0.802 | 0.876 | 1.067 | 1.285 | 1.142 | 1.154 | 1.190 | 1.216 |
| Jiangsu | 0.819 | 0.938 | 1.208 | 1.456 | 1.291 | 1.444 | 1.359 | 1.385 |
| Zhejiang | 0.937 | 1.030 | 1.331 | 1.586 | 1.250 | 1.364 | 1.407 | 1.463 |
| Shanghai | 0.841 | 0.985 | 1.415 | 1.608 | 1.345 | 1.385 | 1.473 | 1.530 |
| Guangdong | 1.144 | 1.258 | 1.478 | 1.655 | 1.323 | 1.375 | 1.471 | 1.536 |
| Hainan | 0.823 | 0.799 | 0.881 | 1.035 | 0.884 | 1.019 | 0.962 | 0.985 |
| Fujian | 0.918 | 1.026 | 1.233 | 1.385 | 1.164 | 1.209 | 1.324 | 1.398 |
| Liaoning | 0.762 | 0.807 | 1.027 | 1.206 | 1.066 | 1.063 | 1.066 | 1.092 |
| Shanxi | 0.625 | 0.653 | 0.846 | 0.988 | 0.842 | 0.910 | 0.940 | 0.571 |
| Henan | 0.784 | 0.738 | 0.879 | 1.102 | 1.013 | 1.040 | 1.094 | 1.122 |
| Anhui | 0.791 | 0.820 | 0.947 | 1.157 | 1.037 | 1.062 | 1.107 | 1.144 |
| Hubei | 0.725 | 0.708 | 0.909 | 1.092 | 0.955 | 1.016 | 1.109 | 1.180 |
| Jiangxi | 0.651 | 0.694 | 0.856 | 1.048 | 0.955 | 0.979 | 1.092 | 1.147 |
| Hunan | 0.797 | 0.722 | 0.882 | 1.101 | 0.962 | 0.987 | 1.114 | 1.158 |
| Jilin | 0.613 | 0.696 | 0.819 | 0.998 | 0.958 | 0.972 | 1.006 | 1.031 |
| Heilongjiang | 0.516 | 0.654 | 0.778 | 0.925 | 0.827 | 0.935 | 0.955 | 1.001 |
| Chongqing | 0.819 | 0.856 | 1.098 | 1.297 | 1.085 | 1.180 | 1.209 | 1.301 |
| Sichuan | 0.777 | 0.815 | 0.987 | 1.138 | 1.026 | 1.037 | 1.110 | 1.142 |
| Shaanxi | 0.676 | 0.760 | 0.884 | 0.985 | 0.917 | 1.002 | 1.046 | 1.159 |
| Yunnan | 0.731 | 0.889 | 0.943 | 1.108 | 0.992 | 0.968 | 0.901 | 0.915 |
| Guizhou | 0.687 | 0.759 | 0.856 | 1.053 | 0.959 | 0.953 | 0.934 | 0.961 |
| Guangxi | 0.751 | 0.805 | 0.951 | 1.063 | 1.070 | 1.100 | 1.056 | 1.075 |
| Gansu | 0.653 | 0.728 | 0.789 | 0.976 | 0.853 | 0.800 | 0.902 | 0.929 |
| Qinghai | 0.483 | 0.651 | 0.722 | 0.892 | 0.776 | 0.732 | 0.784 | 0.801 |
| Ningxia | 0.491 | 0.645 | 0.839 | 0.974 | 0.899 | 0.882 | 0.930 | 0.967 |
| Xizang | 0.615 | 0.642 | 0.712 | 0.796 | 0.692 | 0.585 | 0.666 | 0.666 |
| Xinjiang | 0.540 | 0.662 | 0.840 | 0.922 | 0.774 | 0.699 | 0.838 | 0.851 |
| Neimenggu | 0.556 | 0.689 | 0.837 | 1.033 | 0.912 | 0.950 | 0.913 | 0.899 |

**S1 Table. The economic institutional change of provinces and cities in China**
