# Supplementary material for: The spatial correlation of economic institutional change in China and its impact on economic growth: A social network analysis approach
Source: PLoS One. 2024 Oct 22;19(10):e0297354. doi: 10.1371/journal.pone.0297354 (PMC11495625; doi:10.1371/journal.pone.0297354)
Supplement: S2 Table — (DOCX) [file pone.0297354.s004.docx]

**S2 Table. The analysis of centrality of provinces and municipalities across China**

(a)degree centrality

| **provinces** | **1997** | | **2003** | | **2009** | | **2015** | |
| --- | --- | --- | --- | --- | --- | --- | --- | --- |
|  | Out-degree centrality | In-degree centrality | Out-degree centrality | In-degree centrality | Out-degree centrality | In-degree centrality | Out-degree centrality | In-degree centrality |
| Hebei | 4 | 3 | 3 | 2 | 5 | 4 | 5 | 5 |
| Beijing | 6 | 22 | 7 | 26 | 7 | 24 | 6 | 23 |
| Tianjin | 8 | 18 | 5 | 20 | 5 | 22 | 5 | 23 |
| Shandong | 4 | 13 | 4 | 6 | 7 | 19 | 8 | 16 |
| Jiangsu | 2 | 18 | 2 | 17 | 5 | 22 | 4 | 27 |
| Zhejiang | 2 | 17 | 3 | 21 | 4 | 20 | 7 | 19 |
| Shanghai | 6 | 29 | 7 | 28 | 9 | 28 | 8 | 25 |
| Guangdong | 11 | 16 | 11 | 15 | 11 | 14 | 11 | 11 |
| Hainan | 3 | 1 | 4 | 1 | 5 | 1 | 6 | 1 |
| Fujian | 4 | 7 | 6 | 1 | 7 | 2 | 7 | 6 |
| Liaoning | 5 | 2 | 4 | 3 | 5 | 3 | 8 | 2 |
| Shanxi | 6 | 2 | 3 | 2 | 6 | 4 | 6 | 4 |
| Henan | 7 | 5 | 6 | 5 | 6 | 7 | 6 | 10 |
| Anhui | 3 | 6 | 4 | 5 | 3 | 8 | 3 | 9 |
| Hubei | 5 | 2 | 6 | 2 | 7 | 4 | 7 | 5 |
| Jiangxi | 5 | 3 | 6 | 4 | 7 | 5 | 8 | 6 |
| Hunan | 6 | 2 | 5 | 2 | 7 | 3 | 7 | 4 |
| Jilin | 5 | 2 | 5 | 1 | 6 | 2 | 6 | 2 |
| Heilongjiang | 5 | 1 | 5 | 0 | 8 | 2 | 8 | 2 |
| Chongqing | 7 | 1 | 7 | 1 | 9 | 2 | 8 | 3 |
| Sichuan | 8 | 1 | 6 | 1 | 9 | 2 | 9 | 2 |
| Shaanxi | 7 | 0 | 7 | 0 | 8 | 0 | 8 | 1 |
| Yunnan | 7 | 1 | 6 | 1 | 7 | 1 | 9 | 2 |
| Guizhou | 7 | 2 | 7 | 2 | 8 | 2 | 9 | 2 |
| Guangxi | 6 | 2 | 5 | 1 | 6 | 1 | 7 | 1 |
| Gansu | 7 | 0 | 7 | 0 | 8 | 1 | 10 | 3 |
| Qinghai | 7 | 0 | 6 | 0 | 9 | 0 | 9 | 1 |
| Ningxia | 7 | 0 | 6 | 0 | 7 | 0 | 8 | 0 |
| Xizang | 8 | 0 | 6 | 0 | 8 | 0 | 8 | 0 |
| Xinjiang | 6 | 0 | 6 | 0 | 8 | 0 | 8 | 0 |
| Neimenggu | 4 | 2 | 3 | 1 | 6 | 10 | 5 | 9 |

(b)closeness centrality

| **provinces** | **1997** | | **2003** | | **2009** | | **2015** | |
| --- | --- | --- | --- | --- | --- | --- | --- | --- |
|  | Out-closeness centrality | In-closeness centrality | Out-closeness centrality | In-closeness centrality | Out-closeness centrality | In-closeness centrality | Out-closeness centrality | In-closeness centrality |
| Hebei | 109 | 69 | 110 | 62 | 101 | 60 | 117 | 60 |
| Beijing | 102 | 42 | 117 | 34 | 108 | 37 | 125 | 38 |
| Tianjin | 97 | 48 | 120 | 42 | 115 | 40 | 126 | 38 |
| Shandong | 101 | 47 | 109 | 54 | 99 | 41 | 112 | 44 |
| Jiangsu | 120 | 42 | 117 | 43 | 100 | 38 | 112 | 33 |
| Zhejiang | 120 | 43 | 107 | 39 | 101 | 40 | 108 | 42 |
| Shanghai | 101 | 31 | 102 | 32 | 94 | 32 | 105 | 35 |
| Guangdong | 88 | 52 | 90 | 59 | 86 | 60 | 96 | 63 |
| Hainan | 98 | 81 | 94 | 88 | 89 | 89 | 101 | 92 |
| Fujian | 104 | 73 | 97 | 87 | 92 | 60 | 108 | 75 |
| Liaoning | 88 | 170 | 108 | 61 | 99 | 64 | 97 | 198 |
| Shanxi | 107 | 70 | 110 | 62 | 100 | 60 | 114 | 61 |
| Henan | 91 | 59 | 107 | 55 | 100 | 53 | 114 | 50 |
| Anhui | 119 | 54 | 109 | 57 | 115 | 52 | 123 | 51 |
| Hubei | 93 | 59 | 92 | 60 | 86 | 58 | 105 | 58 |
| Jiangxi | 100 | 58 | 91 | 58 | 86 | 57 | 98 | 57 |
| Hunan | 91 | 79 | 93 | 86 | 86 | 59 | 99 | 61 |
| Jilin | 96 | 170 | 107 | 90 | 98 | 92 | 99 | 198 |
| Heilongjiang | 96 | 171 | 102 | 210 | 96 | 92 | 97 | 198 |
| Chongqing | 89 | 109 | 90 | 116 | 83 | 115 | 99 | 115 |
| Sichuan | 89 | 81 | 92 | 88 | 83 | 88 | 97 | 90 |
| Shaanxi | 87 | 180 | 86 | 210 | 80 | 210 | 90 | 194 |
| Yunnan | 90 | 81 | 92 | 88 | 86 | 89 | 96 | 90 |
| Guizhou | 89 | 80 | 90 | 87 | 84 | 88 | 96 | 90 |
| Guangxi | 91 | 80 | 93 | 88 | 87 | 89 | 99 | 92 |
| Gansu | 87 | 180 | 86 | 210 | 80 | 204 | 82 | 192 |
| Qinghai | 87 | 180 | 87 | 210 | 74 | 210 | 83 | 194 |
| Ningxia | 87 | 180 | 87 | 210 | 94 | 210 | 85 | 210 |
| Xizang | 85 | 180 | 87 | 210 | 80 | 210 | 93 | 210 |
| Xinjiang | 88 | 180 | 87 | 210 | 80 | 210 | 94 | 210 |
| Neimenggu | 109 | 70 | 110 | 63 | 100 | 55 | 126 | 57 |

(c)betweenness centrality

| **provinces** | **1997** | **2003** | **2009** | **2015** |
| --- | --- | --- | --- | --- |
|  | betweenness centrality | betweenness centrality | betweenness centrality | betweenness centrality |
| Hebei | 0.536 | 4.807 | 5.323 | 5.625 |
| Beijing | 76.110 | 141.217 | 126.504 | 43.495 |
| Tianjin | 49.943 | 29.067 | 23.128 | 26.590 |
| Shandong | 27.464 | 7.291 | 25.604 | 24.717 |
| Jiangsu | 3.283 | 4.075 | 37.970 | 57.628 |
| Zhejiang | 2.950 | 31.920 | 24.002 | 40.928 |
| Shanghai | 143.074 | 178.296 | 151.946 | 90.103 |
| Guangdong | 229.262 | 218.460 | 186.588 | 175.677 |
| Hainan | 0.000 | 0.624 | 0.706 | 0.532 |
| Fujian | 8.781 | 5.840 | 1.708 | 5.189 |
| Liaoning | 3.800 | 30.971 | 57.481 | 0.926 |
| Shanxi | 1.286 | 4.807 | 7.951 | 10.982 |
| Henan | 63.743 | 16.277 | 16.659 | 27.712 |
| Anhui | 2.283 | 12.308 | 8.551 | 9.997 |
| Hubei | 30.438 | 47.035 | 53.640 | 6.204 |
| Jiangxi | 44.533 | 106.163 | 57.529 | 97.397 |
| Hunan | 11.521 | 4.474 | 28.530 | 46.548 |
| Jilin | 1.000 | 0.500 | 0.333 | 0.000 |
| Heilongjiang | 0.000 | 0.000 | 1.833 | 1.250 |
| Chongqing | 0.500 | 0.000 | 1.000 | 3.167 |
| Sichuan | 3.838 | 1.058 | 16.756 | 12.580 |
| Shaanxi | 0.000 | 0.000 | 0.000 | 0.000 |
| Yunnan | 3.505 | 1.058 | 1.176 | 12.256 |
| Guizhou | 32.207 | 30.058 | 15.176 | 12.256 |
| Guangxi | 8.407 | 0.724 | 0.953 | 1.215 |
| Gansu | 0.000 | 0.000 | 0.000 | 18.000 |
| Qinghai | 0.000 | 0.000 | 0.000 | 0.000 |
| Ningxia | 0.000 | 0.000 | 0.000 | 0.000 |
| Xizang | 0.000 | 0.000 | 0.000 | 0.000 |
| Xinjiang | 0.000 | 0.000 | 0.000 | 0.000 |
| Neimenggu | 0.536 | 1.971 | 6.951 | 5.027 |
