# Supplementary material for: The spatial correlation of economic institutional change in China and its impact on economic growth: A social network analysis approach
Source: PLoS One. 2024 Oct 22;19(10):e0297354. doi: 10.1371/journal.pone.0297354 (PMC11495625; doi:10.1371/journal.pone.0297354)
Supplement: S3 Table — (DOCX) [file pone.0297354.s005.docx]

| **Year** | **plates** | **Density matrix** | | | | **Image Matrix** | | | |
| --- | --- | --- | --- | --- | --- | --- | --- | --- | --- |
|  |  | PlateⅠ | PlateⅡ | PlateⅢ | PlateⅣ | PlateⅠ | PlateⅡ | PlateⅢ | PlateⅣ |
| 1997 | PlateⅠ | 0.200 | 0.010 | 0.833 | 0.233 | 1 | 0 | 1 | 1 |
|  | PlateⅡ | 0.000 | 0.011 | 0.608 | 0.847 | 0 | 0 | 1 | 1 |
|  | PlateⅢ | 0.333 | 0.078 | 1.000 | 0.133 | 1 | 0 | 1 | 0 |
|  | PlateⅣ | 0.000 | 0.212 | 0.067 | 0.300 | 0 | 1 | 0 | 1 |
| 2003 | PlateⅠ | 0.089 | 0.000 | 1.000 | 0.375 | 0 | 0 | 1 | 1 |
|  | PlateⅡ | 0.015 | 0.220 | 0.824 | 0.941 | 0 | 1 | 1 | 1 |
|  | PlateⅢ | 0.625 | 0.000 | 1.000 | 0.000 | 1 | 0 | 1 | 0 |
|  | PlateⅣ | 0.094 | 0.221 | 0.000 | 0.417 | 0 | 1 | 0 | 1 |
| 2009 | PlateⅠ | 0.300 | 0.000 | 0.750 | 0.450 | 1 | 0 | 1 | 1 |
|  | PlateⅡ | 0.000 | 0.029 | 0.722 | 0.944 | 0 | 0 | 1 | 1 |
|  | PlateⅢ | 0.450 | 0.097 | 0.583 | 0.125 | 1 | 0 | 1 | 1 |
|  | PlateⅣ | 0.000 | 0.319 | 0.063 | 0.417 | 0 | 1 | 0 | 1 |
| 2015 | PlateⅠ | 0.086 | 0.010 | 0.883 | 0.667 | 0 | 0 | 1 | 1 |
|  | PlateⅡ | 0.000 | 0.071 | 0.464 | 0.800 | 0 | 0 | 1 | 1 |
|  | PlateⅢ | 0.133 | 0.250 | 0.583 | 0.100 | 0 | 1 | 1 | 0 |
|  | PlateⅣ | 0.040 | 0.714 | 0.050 | 0.400 | 0 | 1 | 0 | 1 |

**S3 Table. Block model analysis**
